# Coenzyme Q_10_ defects may be associated with a deficiency of Q_10_-independent mitochondrial respiratory chain complexes

**DOI:** 10.1186/s40659-015-0065-0

**Published:** 2016-01-08

**Authors:** Konstantina Fragaki, Annabelle Chaussenot, Jean-François Benoist, Samira Ait-El-Mkadem, Sylvie Bannwarth, Cécile Rouzier, Charlotte Cochaud, Véronique Paquis-Flucklinger

**Affiliations:** School of Medicine, IRCAN, UMR CNRS 7284/INSERM U1081/UNS, Nice Sophia-Antipolis University, 28 av de Valombrose, 06107 Nice Cedex 2, France; Department of Medical Genetics, Nice Teaching Hospital, National Centre for Mitochondrial Diseases, Nice, France; Department of Biochemistry, Robert Debre Hospital, Paris, France

**Keywords:** Mitochondrial disease, CoQ_10_ deficiency, Respiratory chain, Spectrophotometry, LC-MSMS

## Abstract

**Background:**

Coenzyme Q_10_ (CoQ_10_ or ubiquinone) deficiency can be due either to mutations in genes involved in CoQ_10_ biosynthesis pathway, or to mutations in genes unrelated to CoQ_10_ biosynthesis. CoQ_10_ defect is the only oxidative phosphorylation disorder that can be clinically improved after oral CoQ_10_ supplementation. Thus, early diagnosis, first evoked by mitochondrial respiratory chain (MRC) spectrophotometric analysis, then confirmed by direct measurement of CoQ_10_ levels, is of critical importance to prevent irreversible damage in organs such as the kidney and the central nervous system. It is widely reported that CoQ_10_ deficient patients present decreased quinone-dependent activities (segments I + III or G3P + III and II + III) while MRC activities of complexes I, II, III, IV and V are normal. We previously suggested that CoQ_10_ defect may be associated with a deficiency of CoQ_10_-independent MRC complexes. The aim of this study was to verify this hypothesis in order to improve the diagnosis of this disease.

**Results:**

To determine whether CoQ_10_ defect could be associated with MRC deficiency, we quantified CoQ_10_ by LC-MSMS in a cohort of 18 patients presenting CoQ_10_-dependent deficiency associated with MRC defect. We found decreased levels of CoQ_10_ in eight patients out of 18 (45 %), thus confirming CoQ_10_ disease.

**Conclusions:**

Our study shows that CoQ_10_ defect can be associated with MRC deficiency. This could be of major importance in clinical practice for the diagnosis of a disease that can be improved by CoQ_10_ supplementation.

## Background

Coenzyme Q_10_ (CoQ_10_ or ubiquinone) is a lipid-soluble component of the mitochondrial inner membrane that plays a central role in mitochondrial respiratory chain (MRC) function, as electrons carrier from complexes I and II to complex III, thus participating in ATP production [1].

CoQ_10_ deficiency encompasses several clinical phenotypes such as encephalomyopathy, severe infantile multisystemic disease, cerebellar ataxia, isolated myopathy or nephrotic syndrome [2]. CoQ_10_ deficiency can be primary, due to mutations in genes involved in CoQ_10_ biosynthesis or secondary, due to mutations in genes unrelated to CoQ_10_ biosynthesis [3]. Secondary CoQ_10_ deficiency has been described in patients with mitochondrial DNA (mtDNA) mutations or deletions, with mtDNA depletion syndrome (MDS) [4–6] and in patients with mutations in *APTX* [7], *ETFDH* [8, 9], *BRAF* [10], *ACADVL* or *NPC* genes [11]. CoQ_10_ defect is the only oxidative phosphorylation (OXPHOS) disorder that can be clinically improved after oral CoQ_10_ supplementation with limitation of neurological and renal manifestations, amelioration of muscular symptoms and attenuation of histological alterations. Early treatment is crucial to prevent irreversible damage in organs such as the kidney and the central nervous system [12–14]. Reduced activities of CoQ_10_-dependent enzymes by spectrophotometric analysis (segments I + III or G3P + III and II + III) are evocative of CoQ_10_ deficiency but direct measurement of CoQ_10_ levels is the most reliable test for diagnosis [15]. It is widely reported in the literature that, in patients with CoQ_10_ deficiency, enzymatic activities of MRC complexes I, II, III, IV, V are normal [16]. In a previous report, we described an 11-year-old boy presenting with a propionic acidemia who succumbed to acute heart failure in the absence of decompensation of his metabolic condition. Spectrophotometric analysis in liver identified CoQ_10_-dependent activities deficiency that was associated with MRC enzymatic defect. Secondary CoQ_10_ deficiency was likely involved in the development of heart complications in this child and we hypothesized that a CoQ_10_-defect may be associated with MRC deficiency [17]. The aim of this study was to verify this hypothesis in order to improve the diagnosis of this disease.

Over a 6-year period, we analyzed by spectrophotometry 700 tissue samples from 495 patients in whom a mitochondrial disease was suspected. Isolated CoQ_10_-dependent activity deficiency led to identification of CoQ_10_ disease in eight cases. Eighteen patients presented CoQ_10_-dependent enzymatic deficiency associated with MRC defect by spectrophotometry in muscle or in fibroblasts. In order to validate our original observation and to establish if CoQ_10_ quantitative defect may be associated with multiple MRC enzymatic deficiency, we measured CoQ_10_ in this group of 18 patients. We found decreased CoQ_10_ levels by liquid chromatography coupled with tandem mass spectrometry detection (LC-MSMS) in eight patients out of 18 (45 %), thus confirming CoQ_10_ disease and its association with MRC enzymatic deficiency. Furthermore, CoQ_10_ disease cannot be ruled out in all other patients insofar as the quantitative assay could not always be performed in the affected tissue.

## Results

### Description of patients involved in the study

We studied 18 patients, including 10 males and eight females, ranging in age from day 1 to 76 years. Clinical presentations were very heterogeneous (Table 1). The age at onset of the disease was highly variable, ranging from (i) neonatal forms (seven cases with severe phenotypes), (ii) onset before 1 year of age (four cases with either Leigh syndrome or epileptic encephalopathy), (iii) childhood-onset (four cases including two myopathic forms and two complex phenotypes) to (iv) adult-onset (three cases with two myopathic presentations and one cerebellar ataxia). The 18 patients were divided into two different groups according to molecular results.

**Table 1:** Clinical phenotypes of patients presenting CoQ_10_-dependent enzymatic deficiency associated with MRC defect

Patient	Tissue	Sex	Age at biopsy	Age of onset	Heredity	Familial history	Neurological symptoms	Muscular symptoms		Other symptoms	Muscle histology	Enzymology	Diagnosis or molecular analyses
*Patients with molecular diagnosis*													
P01^a^	Fibroblasts	M	D1	Neonatal	Recessive	Affected brother				Neonatal polyvisceral failure	Not done	Cx IV deficiency; segments II + III and G3P + III reduction	*COQ2*: homozygous mutation (c.437G > A; p.Ser146Asn)
P02	Muscle	M	54y	25y	Sporadic	No	Brain MRI: mild atrophy and lacunar strokes		CPEO	T2DM, hepatic steatosis, dyslipidemia	RRF (5–10 %) and Cox-fibers	Cxes I, II, IV and V deficiency; segments I + III and II + III reduction	Large-scale deletion of mtDNA
P03^a^	Fibroblasts	M	D1	Neonatal	*de novo*	No	Hypotonia, epilepsy and diffuse brain lesions			Neonatal polyvisceral failure: respiratory distress, hepatic failure, hypertrophic CMP, lactic acidosis ++	Not done	Cxes II and III deficiency; segments II + III and G3P + III reduction	*MT*-*CYB*: heteroplasmic mtDNA mutation (m.15635T > C; p.Ser297Pro)
P04^a^	Fibroblasts	M	15y	11y	Recessive	No	Ataxic sensory axonal neuropathy		CPEO		RRF and Cox-fibers (40 %)	Cxes I, II, III and IV deficiency; segments II + III and G3P + III reduction	SANDO with multiple mtDNA deletions and homozygous mutation in *POLG*: (c.911T > G; p.Leu304Arg)
P05	Muscle	F	54y	45y	Recessive	No	Ataxic sensory axonal neuropathy		CPEO		Lipid accumulation, RRF and Cox-fibers (20 %)	Cxes I, II, III, IV and V deficiency; segments I + III and II + III reduction	SANDO with multiple mtDNA deletions and compound heterozygous mutations in *POLG*: (c.752C > T/c.2452G > A; p.Thr251Ile/p.Gly848Ser)
P06^a^	Fibroblasts	M	D1	Neonatal	Recessive	No	Encephalopathy and hypotonia			Severe lactic acidosis, methylmalonic aciduria	Not done	Cxes II, III and IV deficiency; segments II + III and G3P + III reduction	*SUCLG1*: compound heterozygous mutations c.97 + 3G > C/c.509C > G (p.Pro170Arg)
P07	Muscle	F	18y	4y	Recessive	Blindness in paternal family			Bilateral ptosis, proximal myopathy, dysphonia, dysphagia, exercice intolerance	Retinitis pigmentosa, cyclic vomiting, hyperCPKemia	Lipid accumulation	Cxes I and III deficiency; segments I + III and II + III reduction	MADD with mutations in *ETFDH*
P08	Muscle	F	4 m	4 m	Recessive	Affected sister	Encephalopathy with refractory migrating partial seizures				Lipid accumulation	Cx I, II, III and IV deficiency; segments I + III and II + III reduction	Malignant migrating partial seizures with compound heterozygous mutations in *TBC1D24*: (c.468C > A/c.686C > T; p.Cys156X/p.Phe229Ser)
P09	Fibroblasts	M	D1	Neonatal	Recessive	No	Hypotonia			Hypertrophic CMP, dysmorphic, hepatic cytolysis, hypospadia	Glycogenic accumulation	Cxes II, III, IV and V deficiency; segments II + III and G3P + III reduction	CDG syndrome type Iq : homozygous mutation in *SRD5A3* : (c.620T > G; p.Met207Arg)
P10^a^	Fibroblasts	M	3 m	Neonatal	*de novo*	No	Hypotonia, epilepsy, dysphagia			Dilated CMP, aortic dilatation	Glycogenic accumulation	Cxes III and IV deficiency; segments II + III and G3P + III reduction	1p36 deletion syndrome
*Patients with no molecular diagnosis*													
P11	Muscle	M	76y	Adult	?	No	Cerebellar ataxia				2 RRF and Cox- fibers (20-30 %)	Cx IV deficiency; segments I + III and II + III reduction	Multiple mtDNA deletions
P12^a^	Fibroblasts	F	7 m	6 m	?	No	Leigh syndrome				Not done	Cx II deficiency; segments II + III and G3P + III reduction	mtDNA depletion, absence of mtDNA and *POLG, SUCLA2, TK2* mutation
P13^a^	Muscle	F	41y	Childhood	Recessive	Consanguinity	Spastic tetraparesis, chorea, mental retardation		Myopathy	Glaucoma, cataract, lactic acidosis	RRF ++	Cx I deficiency; segments I + III and II + III reduction	Absence of mtDNA and *POLG, OPA1, OPA3* mutation
P14	Muscle	F	33y	6 m	?	No	Epilepsy, spastic diplegia, dystonia, dyskinesia, tremor				1 Cox-fiber	Cxes III and V deficiency; segments I + III and II + III reduction	Absence of mtDNA and *POLG, TTC19, DYT5* mutation
P15	Muscle	F	28 y	Childhood	Recessive	Affected siblings	Encephalopathy, mental retardation				Normal	Cxes II, III and V deficiency; segments I + III and II + III reduction	Absence of mtDNA mutation
P16	Fibroblasts	M	9y	Infancy	?	No	Psychomotor retardation, behavior disorders, dystonia, dyspraxia and basal ganglia involvement at brain MRI (Leigh)				Normal	Cxes II and III deficiency; segments II + III and G3P + III reduction	Absence of mtDNA mutation
P17	Fibroblasts	F	D3	D2	?	No				Unexplained severe respiratory failure	Normal	Cxes II, III deficiency; segments II + III and G3P + III reduction	Absence of mtDNA mutation
P18	Fibroblasts	M	2y	D18	?	No	Encephalopathy with refractory epilepsy			Microcephaly	Normal	Cxes III and IV deficiency; segments II + III and G3P + III reduction	Absence of mtDNA mutation

*M* male, *F* female, *D* day, *m* month, *y* year, *CPK* Creatine PhosphoKinase, *CPEO* Chronic Progressive External Ophthalmoplegia, *T2DM* Type 2 Diabetes Mellitus, *CMP* CardioMyoPathy, *RRF* Ragged Red Fibers, *Cox* cytochrome *c* oxydase, *cx* complex, *mtDNA* mitochondrial DNA, *SANDO* Sensory Ataxia Neuropathy Dysarthria and Ophthalmoplegia, *MADD* Multiple Acyl-CoA Dehydrogenation Deficiency, *CDG* Carbohydrate-Deficient Glycoprotein

^a^Patient deceased

The first group included 10 patients with identified mutations in responsible genes (Table 1). Patient P01 presented a severe neonatal multisystemic disease secondary to a homozygous missense mutation in the *CoQ2* gene [18]. Spectrophotometric analysis in fibroblasts revealed a CoQ_10_-dependent activities defect (segments II + III and G3P + III reduction) associated with a complex IV deficiency (Table 2). Six patients (P02–P07) presented a mitochondrial disease or dysfunction secondary either to mtDNA abnormalities (P02 and P03) or to mutations in nuclear genes (P04–P07). Patient P02 had a large heteroplasmic mtDNA deletion responsible for Kearns–Sayre syndrome and patient P03 presented with a severe neonatal polyvisceral failure secondary to a heteroplasmic mtDNA mutation in the *MT*-*CYB* gene. Patients P04 and P05 presented with sensory ataxia neuropathy dysarthria and ophthalmoplegia (SANDO) phenotype associated with recessive mutations in *POLG.* Patient P06 had a neonatal encephalopathy with lactic acidosis and mild methylmalonic aciduria linked to mutations in the *SUCLG1* gene. P07 had a diagnosis of multiple acyl-CoA dehydrogenation deficiency (MADD) with *ETFDH* mutation. The last three patients in the first group presented malignant migrating partial seizures with mutations in *TBC1D24* (P08), CDG syndrome type Iq with *SRD5A3*-*CDG* mutations (P09) and 1p36 deletion syndrome (P10). Patients P02–P10 had a CoQ_10_-dependent activities deficiency (segments I + III or G3P + III and II + III reduction) associated with a multiple MRC defect in muscle or in fibroblasts (Table 2).

**Table 2:** Biochemical analysis of patient fibroblasts and muscle biopsies

OXPHOS activities (spectrophotometry)	I	II	III	IV	V	G3P + III	II + III	CS	CoQ10 quantity (LC-MSMS)	CoQ10
*Fibroblast measurements*										
*Control values (nmole/min/mg of proteins)*	*9.0–27.1*	*21.0–54.0*	*62.0–176.2*	*109.9–350.0*	*22.0–46.2*	*6.5–23.0*	*15.0–37.2*	*74.7–161.1*	*Control values (pmole/mg of proteins)*	*43.0–120.8*
P01	11.2	27.7	89.7	*29.2*	33.5	*2.3*	*7.5*	156.2	P01	*1.4*
P03	11.5	*18.5*	*21.3*	177.7	34.1	*4.1*	*12.5*	106.7	P03	65.4
P04	*7.5*	*18.6*	*40.2*	*108.2*	30.5	*4.7*	*8.6*	95.0	P04	*9.7*
P06	11.6	*20.6*	*47.9*	*78.1*	37.6	*5.5*	*10.8*	116.6	P06	*5.9*
P09	12.0	*13.4*	*53.4*	*57.0*	*15.8*	*4.1*	*8.9*	80.9	P09	62.0
P10	13.5	22.9	*54.4*	*65.0*	28.3	*5.4*	*12.0*	148.2	P10	55.9
P12	10.9	*17.6*	76.6	173.3	25.0	*4.4*	*10.1*	102.5	P12	62.1
P16	11.2	*20.7*	*57.4*	134.9	38.3	*6.1*	*14.5*	124.0	P16	*5.7*
P17	12.9	*20.0*	*61.5*	181.7	29.3	*5.6*	*14.7*	130.3	P17	58.1
P18	14.4	22.5	*39.4*	*78.9*	39.3	*5.5*	*13.2*	147.0	P18	58.2
*Muscle biopsy measurements*										
*Control values (nmole/min/mg of proteins)*	*11.0–32.0*	*22.0–65.0*	*109.0–236.0*	*93.0-347.0*	*40.0–89.0*	*14.0–50.0*	*20.0–50.0*	*82.0–234.0*	*Control values (pmole/mg of proteins)*	*17.8 –22.2*
P02	*6.7*	*21.5*	130.4	*56.9*	*32.5*	*7.4*	*10.8*	113.4	P02	*16.0*
P05	*5.7*	*21.2*	*28.9*	*59.4*	*12.7*	*10.9*	*15.2*	122.2	P05	35.5
P07	*4.2*	28.6	*108.8*	170.4	50.0	*10.5*	*16.7*	272.4	P07	25.9
P08	*10.9*	*14.1*	*102.5*	*92.8*	63.3	*10.8*	*17.0*	116.5	P08	*5.7*
P11	25.7	29.7	157.6	*80.2*	45.0	*13.6*	*19.6*	109.9	P11	*6.2*
P13	*7.6*	28.9	112.7	212.7	58.4	*9.4*	*13.4*	192.6	P13	22.4
P14	15.5	26.6	*31.6*	154.5	*32.8*	*13.7*	*13.2*	100.5	P14	22.2
P15	16.2	*20.0*	*92.3*	191.7	*39.8*	*11.9*	*17.5*	86.1	P15	*7.1*

Respiratory chain enzyme activities were measured spectrophotometrically. Results are expressed as absolute values for controls or patients (in nanomoles of substrate per minute per milligram of protein). CoQ_10_ quantity was measured by LC-MSMS. Results are expressed as absolute values for controls or patients (in picomoles per milligram of protein). Abnormal values are shown in italics

*OXPHOS* oxidative phosphorylation; *LC-MSMS* liquid chromatography coupled with tandem mass spectrometry detection

The second group included eight patients suspected of CoQ_10_ deficiency with an absence of molecular diagnosis. Except for individual P11, who developed cerebellar ataxia during adulthood, all patients had an early-onset disease ranging from neonatal period to childhood. They presented severe neurological symptoms including two Leigh syndromes (P12 and P16) and one child had an unexplained severe respiratory failure at birth (P17). In the second group, all patients presented a CoQ_10_-dependent enzymatic deficiency associated with MRC defect in muscle or in fibroblasts (Table 2).

### Confirmation of CoQ_10_disease in eight patients by CoQ_10_ quantification

Quantitative analysis of CoQ_10_ in muscle or fibroblasts showed that eight patients presented CoQ_10_ content below normal values (Table 2). CoQ_10_ defect was found in five patients out of 10 in the first group and in three patients out of eight in the second group. CoQ_10_-deficient individuals were six males and two females, ranging in age from day 1 to 76 years. The age of onset was highly variable, ranging from neonatal forms to diseases appearing after 25 years of age, although six patients had childhood onset. One patient (P01) presented a polyvisceral failure at birth and all the others had neurological symptoms either isolated or combined with muscular and/or other signs. In the first group, the very low CoQ_10_ level observed in the fibroblasts of patient P01 confirmed the primary CoQ_10_ defect associated with the c.437G > A homozygous missense mutation (p.Ser146Asn) in the *CoQ2* gene, involved in CoQ_10_ biosynthesis [18]. In the four other patients in the same group, CoQ_10_ defect was clearly secondary because the responsible genes were unrelated to CoQ_10_ biosynthesis. Three patients had a mitochondrial disease linked to a large mtDNA deletion (patient P02) or to mutations in *POLG* (patient P04) or *SUCLG1* (patient P06). Patient P08 alone did not have a mitochondrial disease, her encephalopathy with refractory malignant migrating partial seizures being linked to mutations in the *TBC1D24* gene. In the second group, low CoQ_10_ levels were found in three patients with no molecular diagnosis. Two patients were strongly suspected of having a mitochondrial disease: patient P11, who had a cerebellar ataxia with 20–30 % of COX-negative fibers and multiple mtDNA deletions in muscle, and patient P16 who presented with a Leigh syndrome. The last patient (P15) had an encephalopathy with intellectual disability but no histological sign of mitochondrial myopathy.

## Discussion

While primary CoQ_10_ defects are rare, secondary defects have been observed in various pathologies. In a previous work, we suspected for the first time a secondary CoQ_10_ defect in a child with propionic acidemia, who succumbed to acute heart failure in the absence of decompensation of his metabolic condition [17]. CoQ_10_ deficiency was not evoked at the outset because CoQ_10_-dependent activities deficiency was associated with multiple MRC deficiency in the liver of the patient and it had been widely reported that enzymatic activities of MRC complexes are normal in CoQ_10_ disease [16]. However, it is likely that a secondary CoQ_10_ defect was involved in the development of heart complications leading to the child’s death and that oral CoQ_10_ supplementation would have been able to prevent cardiac failure if results had been obtained before acute clinical aggravation. This hypothesis is supported by a recent study, which describes a successful reversal of propionic acidemia-associated cardiomyopathy after treatment [19]. The child in this case presented with myocardial CoQ_10_ quantitative defect associated with signs of mitochondrial dysfunction such as enlarged mitochondria with atypical cristae and low MRC complex IV activity [19]. Several studies performed on cellular models of CoQ_10_ defect suggested a possible association with mitochondrial dysfunction: *PDSS2* and *COQ9* mutant fibroblasts presented a markedly reduced ATP synthesis and *COQ2* mutant fibroblasts presented a partial defect in ATP synthesis, as well as significantly increased ROS production and oxidation of lipids and proteins [20, 21]. In 2013, Duberley and colleagues established the first pharmacologically-induced CoQ_10_ deficient cellular model in neuroblastoma-derived SH-SY5Y cells by using para-aminobenzoic acid (PABA). They showed that, after PABA treatment, SH-SY5Y cells presented a progressive decrease in the activities of CoQ_10_-dependent II + III segment but also a deficiency in MRC complexes I and IV. They also reported a concomitant decrease in the level of total cellular ATP with an increase of mitochondrial oxidative stress [22]. Lastly, deficiency of complexes I, II, III and/or IV has also been previously reported in association with CoQ_10_ defect in the patient’s fibroblasts, muscle or kidney [8, 11, 18, 23].

Today, in most diagnostic laboratories, a spectrophotometric deficiency in one or several MRC enzymes associated with a decrease in CoQ_10_-dependent activities is not considered to be a sign of a CoQ_10_ disease, leading to a possible under-estimation of the frequency of this disorder. With the aim of achieving a better diagnostic approach, we quantified CoQ_10_ by LC-MSMS in 18 patients presenting a CoQ_10_-dependent enzymatic deficiency associated with a MRC defect by spectrophotometry. CoQ_10_ quantitative analysis in muscle or in fibroblast cells confirmed CoQ_10_ disease in eight patients (45 %). These data show that a primary CoQ_10_ defect can be associated with MRC enzymatic deficiency because patient P01, who carried a deleterious homozygous mutation (c.437G > A; p.Ser146Asn) in the *CoQ2* gene, also presented a complex IV deficiency in muscle. Our data also confirm that a secondary CoQ_10_ defect can be associated with mitochondrial disease. Indeed, three other patients with a low CoQ_10_ level presented a respiratory chain deficiency linked to mtDNA deletion (patient P02) or to mutations in *POLG* and *SUCLG1* genes (patients P04 and P06). Secondary CoQ_10_ defect has already been reported in patients with mitochondrial diseases or dysfunctions including Kearns–Sayre syndrome [24], mtDNA depletion and PEO [5] or mutations in *ETFDH* coding for electrontransferring-flavoprotein dehydrogenase and causing MADD [8, 9]. Secondary CoQ_10_ defect has also been described in non-mitochondrial disorders linked to genes such as *APTX* coding for aprataxin and causing ataxia occulomotor-apraxia [7], *BRAF* coding for serine/threonine-protein kinase B-Raf and causing cardiofaciocutaneous syndrome [10], *ACADVL* causing very long-chain Acyl-CoA dehydrogenase deficiency or *NPC* causing Niemann-Pick-type C disease [11]. Here, we report for the first time a secondary CoQ_10_ defect associated with mutations in the *TBC1D24* gene, leading to malignant migrating partial seizures (Patient P08). The mechanisms linking CoQ_10_ defect and decreased activity of MRC complexes are unknown. Studies in patients with metabolic diseases showed an increase in oxidative stress-markers and a decrease in antioxidant defences [25]. More specifically, ubiquinol depletion in patient tissues may lead to increased reactive oxygen species activity [26] and, since all enzymes of the MRC are susceptible to free radical induced oxidative damage [27], we can hypothesize that CoQ_10_-independent MRC dysfunction may result from a high level of mitochondrial oxidative stress creating an imbalance with the CoQ_10_ antioxidant capacity, as previously evoked [25]. In parallel, a possible reason for a secondary CoQ_10_ defect resulting from a primary MRC deficiency is that the enzymes involved in CoQ_10_ biosynthesis are found in a supercomplex in the inner mitochondrial membrane [28]. We hypothesize that the increased oxidative stress resulting from a primary MRC deficiency may inhibit these enzymes resulting in a secondary CoQ_10_ defect.

## Conclusions

In conclusion, our work highlights the probability that, based on spectrophotometric analysis, the frequency of CoQ_10_ disease is underestimated in routine clinical practice. Several studies, which performed a systematic CoQ_10_ quantification on muscle biopsies from pediatric and adult populations presenting a wide range of clinical phenotypes, also reported an underestimation of CoQ_10_ defects and proposed a systematic evaluation of CoQ_10_ content in all muscle biopsies [5, 29, 30]. However, first-line CoQ_10_ quantification seems difficult to set up as a routine analysis in all diagnosis laboratories. Based on our observations, we suggest that CoQ_10_ quantification be performed in all tissues presenting a spectrophotometric deficiency of CoQ_10_-dependent enzymes, associated or not with MRC defect, regardless of the patient’s age, clinical presentation or molecular diagnosis. This could prove of great value in clinical practice for the diagnosis of a disease that can be improved by CoQ_10_ supplementation.

## Methods

### Patients

All patients were explored in the Reference Centre for Mitochondrial Disease (CHU of Nice, France). Selection of the 18 patients was based on the following inclusion criteria: (1) availability of a muscle sample or fibroblast culture and (2) spectrophotometric deficiency of CoQ_10_-dependent activities (reduction of segments I + III or G3P + III and II + III) associated with MRC defect in muscle or in fibroblasts. The following data were systematically collected: sex, age at biopsy, age of onset, heredity, familial history, clinical presentation, brain MRI, metabolic screening, mitochondrial enzymatic studies, histological and molecular analyses. The age of onset of clinical symptoms ranged from neonatal period to 45 years of age. Blood and tissue samples were obtained after adult patients and parents of affected children had given informed consent.

Patients were divided into two groups (Table 1), according to the results of molecular analysis: (1) individuals with a molecular diagnosis, carrying mutations in mtDNA or in nuclear genes and, (2) individuals with no molecular diagnosis.

### Cell culture

Primary fibroblast cultures were obtained from patient skin punches, using standard procedures, in RPMI medium supplemented with 10 % Fetal Bovine Serum, 45 μg/ml uridine and 275 μg/ml sodium pyruvate. Cultures were incubated at 37 ℃ with 5 % CO_2_.

### OXPHOS spectrophotometric measurements

Enzymatic spectrophotometric measurements of the OXPHOS respiratory chain complexes and citrate synthase were performed at 37 ℃ on muscle crude homogenates or fibroblasts according to standard procedures [31]. Proteins were measured according to Bradford microassay [32] and results were expressed as nmole/min/mg of proteins.

### Coenzyme Q_10_ quantification

Total coenzyme Q_10_ was extracted from tissues and analyzed by reverse phase liquid chromatography separation (column C18 symmetry 150 × 2.1 mm, 3.5 µm, Waters, France) as previously described [33]. Detection and quantification were done by mass spectrometry using an API 3000 tandem mass spectrometer (ABSciex, France) equipped with an APCI source. CoQ_10_ and CoQ_9_ were analyzed in the positive mode using the following m/z 864 → 197 and 796 → 197 transitions. CoQ_9_ was used as internal standard for quantification. External calibration was performed using CoQ_10_ solutions. A stock solution was prepared by dissolving 10 mg of CoQ_10_ in 4 ml of methanol/chloroform (98:2 v/v). This solution was stable for 3 months at −80 °C. The working solutions were prepared daily by diluting the stock solution into methanol to provide a range of 0.05–1 µmol/L. The intra-assay and inter-assay CV’s were, respectively, 5.7 and 6.3 % for a CoQ_10_ concentration of 0.25 µmol/L.

## References

[1] Turunen M, Olsson J, Dallner G (2004). Metabolism and function of coenzyme Q. Biochim Biophys Acta.

[2] Quinzii CM, Hirano M (2010). Coenzyme Q and mitochondrial disease. Dev Disabil Res Rev.

[3] Desbats MA, Lunardi G, Doimo M, Trevisson E, Salviati L (2015). Genetic bases and clinical manifestations of coenzyme Q10 (CoQ 10) deficiency. J Inherit Metab Dis.

[4] Matsuoka T, Maeda H, Goto Y, Nonaka I (1991). Muscle coenzyme Q10 in mitochondrial encephalomyopathies. Neuromuscul Disord.

[5] Sacconi S, Trevisson E, Salviati L, Aymé S, Rigal O, Redondo AG (2010). Coenzyme Q10 is frequently reduced in muscle of patients with mitochondrial myopathy. Neuromuscul Disord.

[6] Montero R, Grazina M, López-Gallardo E, Montoya J, Briones P, Navarro-Sastre A (2013). Coenzyme Q_10_ deficiency in mitochondrial DNA depletion syndromes. Mitochondrion.

[7] Quinzii CM, Kattah AG, Naini A, Akman HO, Mootha VK, DiMauro S (2005). Coenzyme Q deficiency and cerebellar ataxia associated with an aprataxin mutation. Neurology.

[8] Gempel K, Topaloglu H, Talim B, Schneiderat P, Schoser BG, Hans VH (2007). The myopathic form of coenzyme Q10 deficiency is caused by mutations in the electron-transferring-flavoprotein dehydrogenase (ETFDH) gene. Brain.

[9] Liang WC, Ohkuma A, Hayashi YK, López LC, Hirano M, Nonaka I (2009). ETFDH mutations, CoQ10 levels, and respiratory chain activities in patients with riboflavin-responsive multiple acyl-CoA dehydrogenase deficiency. Neuromuscul Disord.

[10] Aeby A, Sznajer Y, Cavé H, Rebuffat E, Van Coster R, Rigal O (2007). Cardiofaciocutaneous (CFC) syndrome associated with muscular coenzyme Q10 deficiency. J Inherit Metab Dis.

[11] Buján N, Arias A, Montero R, García-Villoria J, Lissens W, Seneca S (2014). Characterization of CoQ_10_ biosynthesis in fibroblasts of patients with primary and secondary CoQ_10_ deficiency. J Inherit Metab Dis.

[12] Geromel V, Darin N, Chrétien D, Bénit P, DeLonlay P, Rötig A (2002). Coenzyme Q(10) and idebenone in the therapy of respiratory chain diseases: rationale and comparative benefits. Mol Genet Metab.

[13] Trevisson E, DiMauro S, Navas P, Salviati L (2011). Coenzyme Q deficiency in muscle. Curr Opin Neurol.

[14] García-Corzo L, Luna-Sánchez M, Doerrier C, Ortiz F, Escames G, Acuña-Castroviejo D (2014). Ubiquinol-10 ameliorates mitochondrial encephalopathy associated with CoQ deficiency. Biochim Biophys Acta.

[15] Rustin P, Munnich A, Rötig A (2004). Mitochondrial respiratory chain dysfunction caused by coenzyme Q deficiency. Methods Enzymol.

[16] Rötig A, Mollet J, Rio M, Munnich A (2007). Infantile and pediatric quinone deficiency diseases. Mitochondrion.

[17] Fragaki K, Cano A, Benoist JF, Rigal O, Chaussenot A, Rouzier C (2011). Fatal heart failure associated with CoQ10 and multiple OXPHOS deficiency in a child with propionic acidemia. Mitochondrion.

[18] Diomedi-Camassei F, Di Giandomenico S, Santorelli FM, Caridi G, Piemonte F, Montini G (2007). COQ2 nephropathy: a newly described inherited mitochondriopathy with primary renal involvement. J Am Soc Nephrol.

[19] Baruteau J, Hargreaves I, Krywawych S, Chalasani A, Land JM, Davison JE (2014). Successful reversal of propionic acidaemia associated cardiomyopathy: evidence for low myocardial coenzyme Q10 status and secondary mitochondrial dysfunction as an underlying pathophysiological mechanism. Mitochondrion.

[20] Quinzii CM, López LC, Von-Moltke J, Naini A, Krishna S, Schuelke M (2008). Respiratory chain dysfunction and oxidative stress correlate with severity of primary CoQ10 deficiency. FASEB J..

[21] Quinzii CM, López LC, Gilkerson RW, Dorado B, Coku J, Naini AB (2010). Reactive oxygen species, oxidative stress, and cell death correlate with level of CoQ10 deficiency. FASEB J..

[22] Duberley KE, Abramov AY, Chalasani A, Heales SJ, Rahman S, Hargreaves IP (2013). Human neuronal coenzyme Q10 deficiency results in global loss of mitochondrial respiratory chain activity, increased mitochondrial oxidative stress and reversal of ATP synthase activity: implications for pathogenesis and treatment. J Inherit Metab Dis.

[23] Lalani SR, Vladutiu GD, Plunkett K, Lotze TE, Adesina AM, Scaglia F (2005). Isolated mitochondrial myopathy associated with muscle coenzyme Q10 deficiency. Arch Neurol.

[24] Zierz S, Jahns G, Jerusalem F (1989). Coenzyme Q in serum and muscle of 5 patients with Kearns-Sayre syndrome and 12 patients with ophthalmoplegia plus. J Neurol.

[25] Mc Guire PJ (2009). Parikh A, Diaz GA. Profiling of oxidative stress in patients with inborn errors of metabolism. Mol Genet Metab.

[26] Genova ML, Pich MM, Biondi A, Bernacchia A, Falasca A, Bovina C (2003). Mitochondrial production of oxygen radical species and the role of Coenzyme Q as an antioxidant. Exp Biol Med (Maywood)..

[27] Zhang Y, Marcillat O, Giulivi C, Ernster L, Davies KJ (1990). The oxidative inactivation of mitochondrial electron transport chain components and ATPase. J Biol Chem.

[28] Hsieh EJ, Gin P, Gulmezian M, Tran UC, Saiki R, Marbois BN (2007). Saccharomyces cerevisiae Coq9 polypeptide is a subunit of the mitochondrial coenzyme Q biosynthetic complex. Arch Biochem Biophys.

[29] Montero R, Artuch R, Briones P, Nascimento A, García-Cazorla A, Vilaseca MA (2005). Muscle coenzyme Q10 concentrations in patients with probable and definite diagnosis of respiratory chain disorders. BioFactors.

[30] Miles MV, Miles L, Tang PH, Horn PS, Steele PE, DeGrauw AJ (2008). Systematic evaluation of muscle coenzyme Q10 content in children with mitochondrial respiratory chain enzyme deficiencies. Mitochondrion.

[31] Rustin P, Chretien D, Bourgeron T, Gérard B, Rötig A, Saudubray JM (1994). Biochemical and molecular investigations in respiratory chain deficiencies. Clin Chim Acta.

[32] Bradford MM (1976). A rapid and sensitive method for the quantitation of microgram quantities of protein utilizing the principle of protein-dye binding. Anal Biochem.

[33] Benoist JF, Rigal O, Nivoche Y, Martin C, Biou D, Lombès A (2003). Differences in coenzyme Q10 content in deltoid and quadriceps muscles. Clin Chim Acta.

